# A Mobile Phone App to Stimulate Daily Physical Activity in Patients with Chronic Obstructive Pulmonary Disease: Development, Feasibility, and Pilot Studies

**DOI:** 10.2196/mhealth.4741

**Published:** 2016-01-26

**Authors:** Sigrid NW Vorrink, Helianthe SM Kort, Thierry Troosters, Jan-Willem J Lammers

**Affiliations:** ^1^ Research Group Demand Driven Care Utrecht University of Applied Sciences Utrecht Netherlands; ^2^ Department of Rehabilitation Sciences KU Leuven Leuven Belgium; ^3^ Department of Respiratory Medicine University Medical Center Utrecht Utrecht Netherlands

**Keywords:** telemedicine, mobile phones, chronic obstructive pulmonary disease, motor activity

## Abstract

**Background:**

Patients with chronic obstructive pulmonary disease (COPD) demonstrate reduced levels of daily physical activity (DPA) compared to healthy controls. This results in a higher risk of hospital admission and shorter survival. Performing regular DPA reduces these risks.

**Objective:**

To develop an eHealth intervention that will support patients with COPD to improve or maintain their DPA after pulmonary rehabilitation.

**Methods:**

The design process consisted of literature research and the iterative developing and piloting phases of the Medical Research Council (MRC) model for complex clinical interventions and the involvement of end users. Participants were healthy adults and persons with COPD.

**Results:**

The mobile phone interface met all the set requirements. Participants found that the app was stimulating and that reaching their DPA goals was rewarding. The mean (SD) scores on a 7-point scale for usability, ease of use, ease of learning, and contentment were 3.8 (1.8), 5.1 (1.1), 6.0 (1.6), and 4.8 (1.3), respectively. The mean (SD) correlation between the mobile phone and a validated accelerometer was 0.88 (0.12) in the final test. The idea of providing their health care professional with their DPA data caused no privacy issues in the participants. Battery life lasted for an entire day with the final version, and readability and comprehensibility of text and colors were favorable.

**Conclusions:**

By employing a user-centered design approach, a mobile phone was found to be an adequate and feasible interface for an eHealth intervention. The mobile phone and app are easy to learn and use by patients with COPD. In the final test, the accuracy of the DPA measurement was good. The final version of the eHealth intervention is presently being tested by our group for efficacy in a randomized controlled trial in COPD patients.

## Introduction

Regular physical activity has significant health benefits and contributes to the prevention of non-communicable diseases [1]. Inactivity is estimated to cause 9% of premature mortality worldwide [2]. In older adults, there is strong evidence that regular exercise and participation in physical activity lowers mortality and morbidity [3], and has a significant impact on several psychological and cognitive parameters [4]. Moreover, physical activity has been observed as a behavioral determinant for healthy aging [5].

Physical activity is also a relevant behavioral determinant for patients with chronic diseases, such as chronic obstructive pulmonary disease (COPD), to maintain physical condition [6], and to improve health-related quality of life [7]. COPD is a disabling airway disease with variable extra-pulmonary effects that may contribute to disease severity in individual patients. It mostly affects older adults with a history of tobacco smoke exposure [8]. Patients with COPD demonstrate reduced levels of spontaneous daily physical activity (DPA) compared to healthy controls [9]. This contributes to a higher risk of hospital admission and shorter survival [10].

Pulmonary rehabilitation (PR) generally includes exercise training, education, psychosocial and behavioral interventions, nutritional therapy, and outcome assessment [11,12], and it can help to improve physical capacity. Unfortunately, this effect does not always translate into improved DPA, and when it does, it tends to fade out over time [13-15]. Taking into account the benefits of regular DPA [16], it is important for patients with COPD to improve, or at least to maintain their DPA levels after a rehabilitation program has ended.

Technology-based assistance in health care (eHealth) can help support patients with COPD by improving self-management of the disease. Self-management interventions in patients with COPD have been shown to improve health-related quality of life, to lower the probability of a respiratory-related hospitalization, and to reduce dyspnea [17,18]. It has been postulated that an eHealth intervention might also be beneficial in the self-management of DPA in patients with COPD. An important element for successful implementation of an eHealth intervention is to engage users in the design process because design flaws can affect ease of use, usability, and reliability of the system, which may reduce a user’s willingness to use the intervention [19].

The objective of this study is to develop an eHealth intervention to support patients with COPD in improving or maintaining DPA after PR. We investigate what type of interface is adequate and feasible toward obtaining this objective and scored the resultant eHealth intervention in terms of usability and privacy.

## Methods

### Recruitment

**Figure 1:**
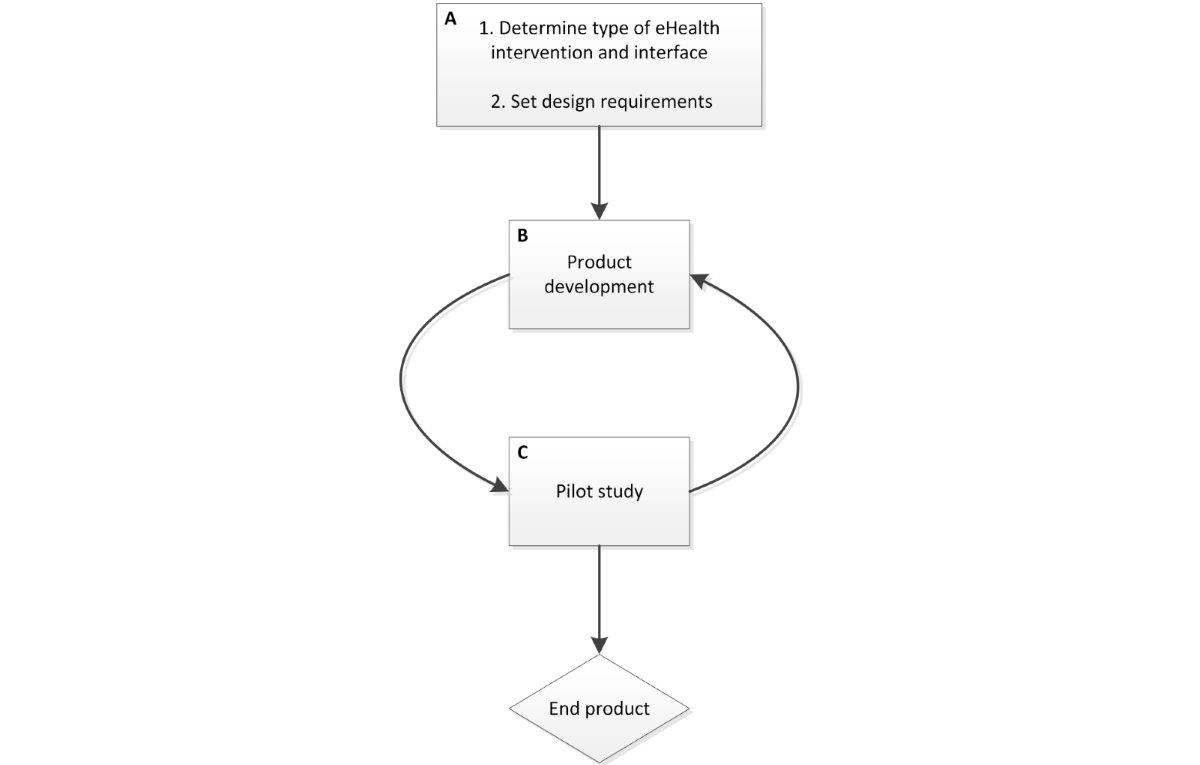
Design process.

The design process was in alignment with the first two phases (developing and piloting) of the Medical Research Council (MRC) model for complex clinical interventions. The key elements of the development and evaluation process of the MRC model were taken into account throughout the design process [20] (Figure 1). This paper primarily focuses on phases A2 through C. Users were defined as persons suffering from COPD, who were aged 40 years or older, living independently, and had completed a rehabilitation program.

### eHealth Intervention and Interface

Based on the literature and our own practice-based experience in the treatment of patients with COPD, the eHealth intervention that we sought to develop had to meet the following requirements: (1) non-obtrusive and easily transportable, (2) objective measurement of DPA, (3) direct feedback and personal DPA, and (4) monitoring and feedback available from a health care professional (HCP).

At the time of this study (2010), several eHealth interventions for physical activity engagement in patients with COPD had been described. They were available in various forms, such as wearable sensors [21], television [22], computers [23], a manual input device [24], and mobile phones [25,26].

As an interface, a smartphone with app capabilities met all set requirements. Although the penetration rate of smartphone use among aging adults was estimated to be low at the time, they were expected to become the majority over the next few years [27,28]. Moreover, mobile touch screens are generally easy for the elderly to use [27], and mobile phones are already equipped with an accelerometer that is both accurate and reliable in measuring and quantifying physical activity in a laboratory setting [29].

Although various apps for mobile phones are available that stimulate engagement in physical activity, none of the apps met all the requirements that are needed to fully address our research goal. Therefore, we decided to develop a new app and an associated website for HCPs. This paper focuses on the development of an app for an eHealth intervention.

We encountered 2 types of apps: those developed for mobile phones running on the interactive operating system (iOS) and those developed for mobiles phones running on the Android operating system. Following a comparison of these 2 operating systems, we opted for the HTC HD2 device (HTC). HTC was chosen as the preferred device based on its higher battery capacity, an absence of restrictions in distributing the app, and its affordable price.

### Pilot Studies

After phases A and B (Figure 1), the product was tested in 3 pilot studies (C1, C2, and C3), and improved through an iterative process. The pilot studies were designed to test the usability of the interface and app, in addition to privacy concerns of the users. The associated website for HCPs was not yet employed in the pilot studies. We also sought to obtain an indication of the accuracy of DPA measurements by the app. Specific sample size recommendations for this type of development and feasibility pilot studies are scarce, as most recommendations are for pilot studies that focus on the feasibility of corresponding RCT studies [30]. The pilot studies were designed to minimize strain on patients with COPD. Therefore, we began the first pilot study with healthy volunteers who had previous mobile phone experience. A subsequent version was then tested in a subset of patients with COPD. Finally, a larger group of patients with COPD were invited to test the final version. We aimed to include 10, 3, and 10 participants in pilot study groups C1, C2, and C3, respectively [31]. The participants in pilot group C1 were recruited from a school, in pilot group C2 they were recruited from a hospital, and in pilot group C3 they were recruited from a rehabilitation center. For inclusion criteria, see Table 1.

**Table 1:** Characteristics of the three pilot studies in study phase C.

Pilot study	Inclusion criteria	Duration of study	Version application
C1	Healthy persons, experience with mobile phones	1 week	Figure 2
C2	Persons suffering from COPD, aged ≥40 years, living independently, and having completed a rehabilitation program	4 days	Figure 3
C3	Persons suffering from COPD, aged ≥40 years, living independently, and having completed a rehabilitation program (same as pilot C2)	3 weeks	Figure 4

The participants received instructions on the functionalities of the mobile phone and app, and information on the course of the study over a training session lasting 1.5 hours. Thereafter, each participant received a HTC Desire A8181 mobile phone with the app installed, and they were given the opportunity to practice, ask questions, and provide feedback. They were instructed to wear the mobile phones in pouches (with various choices for personalization) on their belts. This location was chosen because the best measurements are achieved by positioning the accelerometer as close to the center of gravity as possible [32,33]. They were also instructed to wear accelerometers (BHC0100 Sensewear PRO armband, Body Media, Pittsburgh, US) that had been previously validated in patients with COPD [34-37], on their right upper arms. The armband and mobile phone were worn during waking hours. The participants were instructed to perform their daily activities as usual.

After each study, a group consultation session was held. The sessions started by asking the participants their general impression of the app followed by writing down 3 positive and 3 negative aspects. The most occurring aspects were written on a flip-over and further discussed. The following topics were each discussed for 5 minutes: wearing the mobile phone, using the app, comprehensibility, navigation, future use, and improvements to the app. Sessions were recorded and minutes were made. Afterwards, the sessions were separately summarized by 3 researchers and the main points were taken into consideration for adjustment of the app. Furthermore, the participants were asked to respond to 3 questionnaires (1) the Usefulness, Satisfaction, and Ease of use (USE) questionnaire on usability [38]; (2) the Florida State University (FSU) mobile device feedback preferences scale; and (3) the FSU physiological monitoring privacy scale (inspired by Beach et al [39] and Kwazney et al [40]). Results of the USE questionnaire were compared within and between pilot studies with independent and dependent *t* tests. All of the participants were required to provide signed informed consent prior to the study. Pilot studies were waived from ethics committee approval by the UMC Utrecht Medical Ethical Research Board (number research protocol 10/259). Correlations between the accelerometers on the armbands and the mobile phones were computed by calculating Pearson's correlation coefficient (*r*) in SPSS version 21. The distinctive characteristics of the pilot studies can be found in Table 1.

An additional pilot study, C4, was performed to provide an extra check on DPA measurement accuracy. This was performed with 10 participants who wore the armband and mobile phone for 1 week. Participants met the same inclusion criteria as in pilot studies C2 and C3. These participants did not take part in a consultation round and did not fill out questionnaires since the development of the app was deemed ready at this point.

In pilot study C1, participants were asked to record their daily activities in diaries, including corresponding times of day and durations. In pilot study C3, 3 randomly selected participants wore accelerometers during the first week.

**Figure 2:**
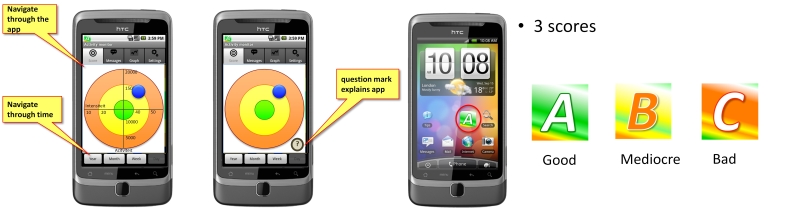
In version 1, the y-axis provides a measure for activity, while the x-axis provides a measure for intensity. The DPA goal is met when the blue ball (representation of current activity status) is kept in the green circle at all times. The widget shows a current status towards reaching a DPA goal (pilot study C1).

## Results

### Setting Design Requirements

A list of design requirements for the eHealth intervention was prepared with respect to the general requirements. Some aspects of the existing apps found during the desk research were also added as requirements. Furthermore, since COPD is inversely related to socioeconomic status and mostly affects older adults [41], special attention was paid to readability and comprehensibility. Focus was put on the mobile phone app for the users (Textbox 1).

**Figure 3:**
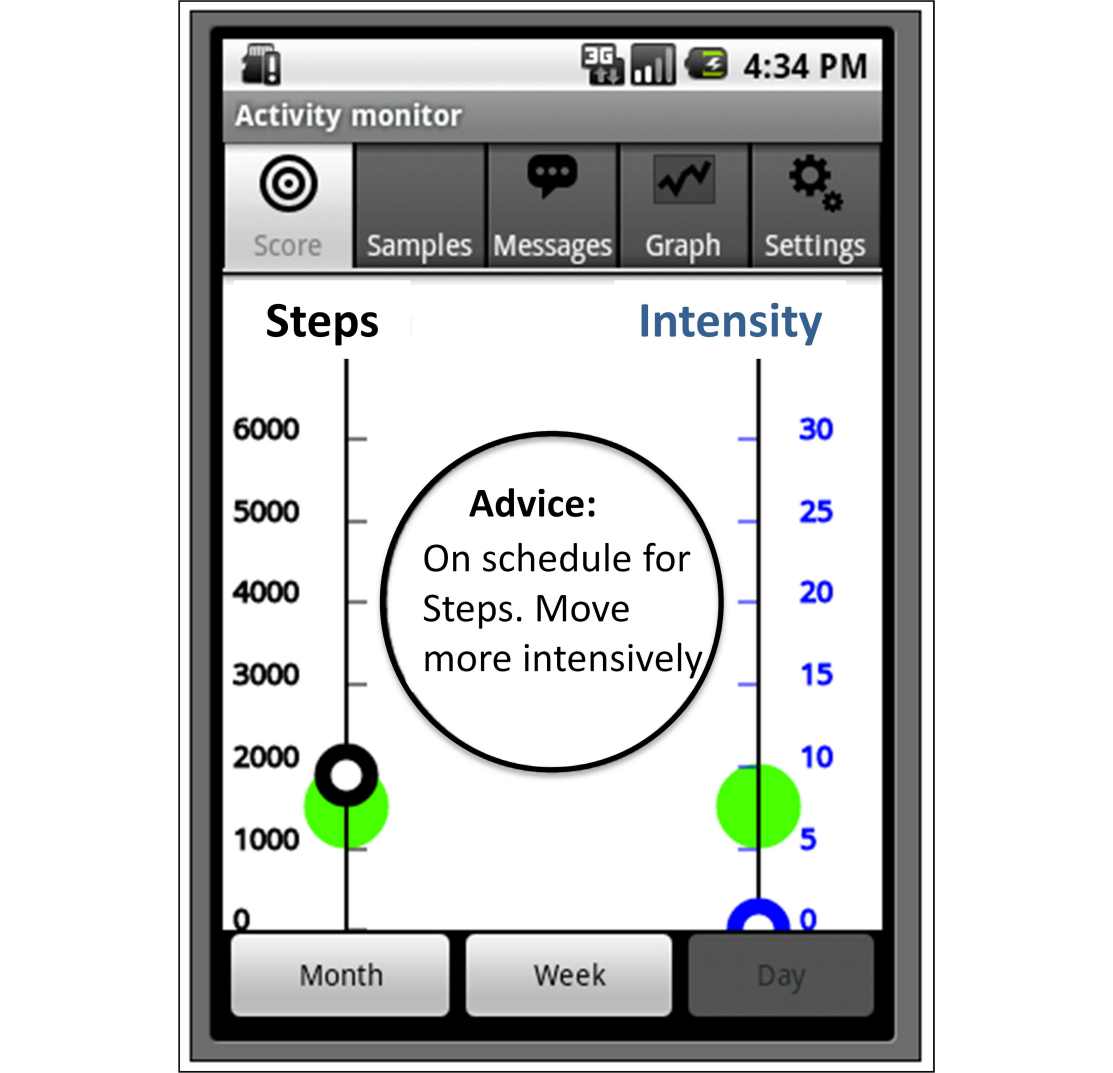
In version 2, the left axis shows amount of steps, while the right axis gives a measure of intensity. The DPA goal is reached when the open circles (representation of current activity status) are kept in the rising green circles at all times (pilot study C2).

**Figure 4:**
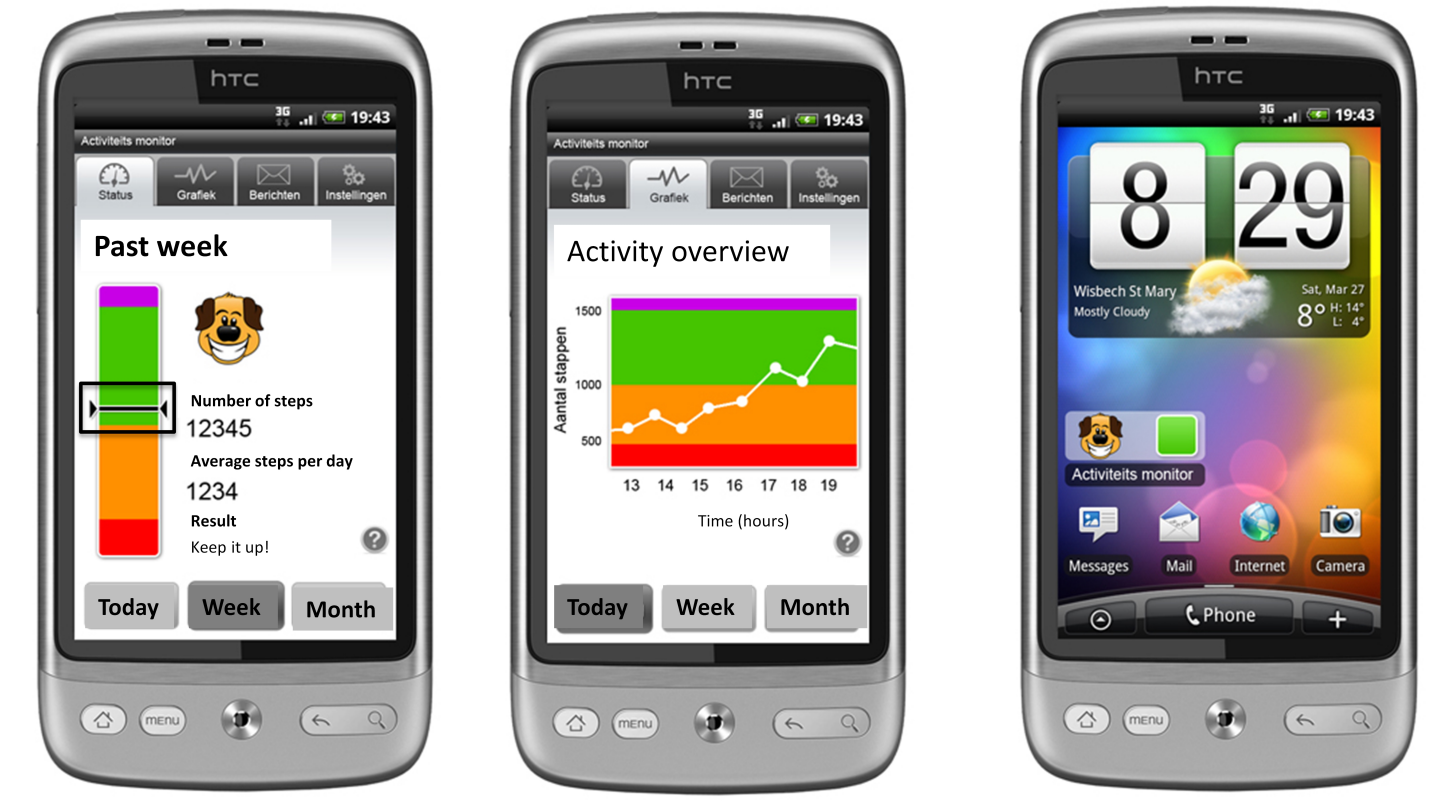
In version 3, the bar on the left side combines amount and intensity of steps. The DPA goal is met when the vertical stripe (representation of current activity status) is kept in the rising rectangle at all times until the green area is reached. Absolute number of steps and current advice on DPA progress are also shown (pilot study C3).

The requirements for the monitoring website for the HCP can be found in Textbox 2. Feedback from HCP on these latter requirements was obtained by consulting with 10 independent respiratory nurses (in a consultation round), and 2 physiotherapists (by phone) who work with COPD patients. Additions to the requirements with regard to privacy and communication were made in response to their feedback.

### Product Development

The app and website were created by a small business enterprise that specializes in developing health care apps. Interactive team work sessions were held during this process. The various designed versions of the app that were tested during the pilot studies are illustrated in Figures 2-4. Communication and multimedia design students from Utrecht University of Applied Sciences were employed to assist in improving the design of the app and the widget after pilot study C2.

### Pilot Studies

A total of 10 participants took part in pilot study C1, 3 in C2, and 7 in C3, of which 1 (10%), 3 (100%), and 4 (57%) were male, respectively. The mean (SD) age of the participants was 21.5 (2.84), 65 (10), and 60.4 (9.4) years in C1, C2, and C3, respectively. The participants were limited in their DPA due to having COPD and were enrolled in a PR program at the time of the study.

The results from the consultation rounds are shown in Multimedia Appendix 1. In pilot study C2, 1 participant (33%, 1/3) was not interested in the intervention; therefore, the results from the consultation round of this group primarily focused on the remaining 2 participants. This participant did fill out the questionnaires. Eleven subjects were recruited to participate in pilot study C3. After the training session, 4 (36%, 4/11) declined to participate due to the degree of expected effort. On day 3 and 7 of pilot study C3, corrected apps were installed due to discovered errors in the algorithm that caused the app to measure too few or no steps.

The results from the USE questionnaire are shown in Table 2. The usability scores for pilot study C1 were significantly lower than ease of use, learning, and contentment scores (*P*<.05 for all). For pilot study C3, usability scores were significantly lower than for ease of learning (*P*<.05). Ease of learning was significantly lower in patients with COPD compared with healthy participants in pilot study C1 (*P*<.004 for C2; *P=*.017 for C3). The feedback preferences questionnaire in general did not provide added insights to the consultation rounds.

**Users' requirements of the mobile phone-based app.**

Software

- Reasonably accurate measurement of DPA
- DPA data are recorded on the mobile phone and available to the user in real time
- Filters out movement produced by riding a car, bus, or train
- Data are available for at least 12 weeks after generation (preferably even longer, such as 6 months to 1 year)
- Data are sent automatically to a secured website for HCP (4-6 times a day)
- Data are only available to users and HCP
- Data are saved when phone runs out of battery
- Data acquisition continues when the mobile phone is in standby mode or is being used for other purposes
- Goal achievement elicits a motivating or complimentary message
- Personal results can be published on social media if desired
- The app uses little energy
- An app-killer is added that can stop all apps except for the intervention
- The app can be used on mobile phones of different brands
- The app can be adjusted in the future

Interface

- DPA is presented in duration, frequency, and intensity
- Data are available in graphs and numbers
- Visual display of progress and goal achievement on screensaver
- Progress is visible in numbers (and percentage until goal is reached)
- Progress is visible based on day, week, and month
- Letters and figures are easily readable (large font and high contrast)
- Navigation is easy and comprehendible; only a few steps are required to reach a desired location
- All text is formulated for persons with low literacy
- The app can be personalized

**Website requirements for the HCP.**

Software

- Data are available for at least 12 weeks after generation (preferably even longer, such as 6 months to 1 year)
- Data are only available to the HCP
- SMS text messages (short message service, SMS) or phone calls can be made from the website
- DPA goals can be adjusted from the website
- Goals can be set based on steps, duration, frequency, and intensity
- Goals are individually adjustable

Interface

- DPA is presented in duration, frequency, and intensity
- Data are available in graphs and numbers
- Overview of the activity status of multiple patients
- Progress of each patient is easily visible in an overview (eg, traffic light colors)
- Individual page for each patient with detailed DPA information

**Table 2:** The mean (SD) scores of the USE questionnaire in the various pilot studies.

Pilot study	Scores, mean (SD)			
	Usability	Ease of use	Ease of learning	Contentment
C1	3.8 (2.0)	5.4 (1.7)	6.6 (0.6)	4.8 (1.7)
C2	3.9 (2.9)	5.1 (2.1)	4.1 (2.9)	5.7 (1.7)
C3	3.7 (2.0)	4.8 (2.2)	5.9 (1.5)	4.4 (1.8)

The correlations between the mobile phones and the armband accelerometers for steps per day are shown in Table 3. The armband of participant 1 (C1) malfunctioned. Participant 2 (C3) only wore the armband for 2 days and was excluded from analysis. The additional pilot study C4 was performed solely to provide an extra check on DPA measurement accuracy. The numbers of valid days for analysis were 8, 4, 8, and 8 for all participants in pilot study C1, C2, C3, and C4, respectively.

**Table 3:** Correlation between mobile phones and armband accelerometers.

Pilot study, *r* ^*a*^	Participant									
	1	2	3	4	5	6	7	8	9	10
C1	N/A	.94^b^	.96^b^	.76^b^	.71^b^	.76^b^	.64	.61	97^b^	.13
C2	.87^b^	.54	.72^b^	N/A	N/A	N/A	N/A	N/A	N/A	N/A
C3	.45	N/A	.67	N/A	N/A	N/A	N/A	N/A	N/A	N/A
C4	.99^b^	.98^b^	.96^b^	.90^b^	.99^b^	.74	.69	.69^b^	.84^b^	.99^b^

^a^Pearson correlation coefficient.

^b^Significant at *P*<.05

## Discussion

### Principal Findings

Engaging patients with COPD in active control over their DPA can work as a preventive measure to prevent functional decline [42]. Therefore, our objective was to develop an eHealth intervention that will help patients with COPD to improve or maintain their DPA after a period of pulmonary rehabilitation. The final product consists of two components (1) a mobile phone app (the focus of this study); and (2) a website for HCPs. The app measures DPA as steps per day, measured by the accelerometer of the mobile phone, and shows this information to the patient via the display of a mobile phone. A physiotherapist can monitor the patient via a secure website where DPA measurements are accessible from all patients. DPA goals can be adjusted and text messages sent to inform and to motivate patients. Furthermore, the website of the intervention can help an HCP work in a more efficient way by monitoring all of their patients at once and enabling them to intervene early on in patients who have trouble maintaining DPA.

#### Use

The mobile phone-based app was found to be easy to learn and use by the participants as well as the patients with COPD. Usability scores were lower than ease of use, learning, and contentment scores. This was significant in pilot study C1 and C3 (for ease of learning). This could be explained, in part, by the fact the app was still in the development phase and still contained some errors, as demonstrated in pilot study C3. Ease of use scores were lower for the patients with COPD, though not significantly. This could be because touch screen pointing performance reduces with age. It is influenced by size, spacing, and location of the target, as well as by size of the device and practice [27,28]. Older people prefer functions that support their declining functional capabilities, and enjoyability is an important determinant of adherence [27]. During the development of the app, attention was paid to all of these aspects. Ease of learning was significantly lower in patients with COPD compared with healthy participants. Proper instruction will greatly influence success in mobile phone usage [43]. Older adults take longer in learning to use mobile phones, and they commit more errors when entering information into mobile phone-based software app [44]. Efforts to overcome these behavioral and attitudinal barriers must include well-designed training that is targeted to older adults to teach mobile phone usage skills as well as creating software with an improved interface and operation [44].

#### Design

The graphic design of the app was adjusted several times to improve use and to provide a better understanding of the DPA data, as well as to accommodate those with low technology literacy. A combination of qualitative and quantitative feedback proved the best fit.

#### Privacy

The key aspect, with respect to privacy, is to give the user control over their data distribution. An important element of the intervention is that an HCP has insight into a patient’s DPA data. This did not pose a problem for the participants in the pilot studies.

#### Measurement of DPA

Distance travelled, cycling, strength training, and the intensity of walking stairs were not properly captured by the app. The first two activities could be added by using GPS data, but this put too much strain on battery life. The accuracy of the measurement varied greatly between participants. Possible reasons for poor correlations include the amount of time spent in a train, bus, or car (participant 7, pilot study C1), unclear diary entries as to whether the mobile phone was worn during exercise (participant 8, pilot study C1), a phone pouch that contained a magnet (participant 10, pilot study C1), using a walker (participant 2, pilot study C2), and using a mobility scooter (all participants of pilot study C3). Using a walker, mobility scooter, or other forms of assistive devices for DPA were added to the exclusion criteria for participants in the randomized controlled trial (RCT). In pilot study C3, the errors in the app in the first week probably also accounted for poor correlations. An additional pilot study (C4) showed a mean (SD) correlation between the armbands and mobile phone accelerometers of *r*=.88 (.12).

### Limitations

During pilot study C3, errors in the algorithm were discovered twice in the distributed app. Although these were swiftly corrected, this could have had a negative impact on the participants’ views of the app.

In pilot study C3, there were 4 (36%, 4/11) dropout participants beforehand due to too much expected effort in learning how to use a mobile phone, and in pilot study C2 there was 1 participant (33%, 1/3) that was not interested in, and did not use, the intervention. This participant had trouble understanding how to use the mobile phone. As mentioned before, proper instruction is key in usage success. More extensive instruction might have improved understanding and prevented dropout. The results of the questionnaires in pilot study C2 may have been negatively influenced by this participant.

Battery life posed a major problem while developing the app. Not all desired options, such as GPS-tracking and continuous measurement, were possible due to limited battery capacity. The “5 minutes on and off” configuration was chosen so the battery would last a whole day, which was deemed important for adherence. With the development of mobile phone technology and accompanying batteries with higher capacity, the app could be adjusted back to continuous measurement, and GPS-tracking could be added.

Using a mobile phone to measure DPA is a good way to obtain objective data on this parameter; however, it is not a highly valid and reliable measurement instrument such as that used in research settings. Additional validated accelerometers would provide improved measurement accuracy of DPA, but it was reasoned that (long-term) adherence to the intervention would benefit from the least amount of devices worn. This app can be useful in obtaining an indication of a patient’s activity outside of a clinical setting. It will provide much more reliable data compared to a patient’s recall [45,46].

### Comparison With Prior Work

A review conducted by Bort-Roig et al [47] evaluated 10 studies that described the accuracy of physical activity data as measured by a mobile phone. The participants were mostly overweight or healthy adults. The studies reported measurement accuracy ranging from 52% to 100% in identifying certain activities and postures (eg, walking or standing). As described, there is room for improvement in DPA measurement accuracy when using a mobile phone accelerometer.

This review also found that physical activity profiles, real-time feedback, social networking, expert consultation, and goal setting were identified as key features that facilitated physical activity engagement. Most of these features are also incorporated in our eHealth intervention.

We found one pilot study that similarly focused on physical activity stimulation in patients with COPD [48]. Their intervention consists of a mobile phone app, website, and separate accelerometer. The participants felt encouraged to be more active. The positive effects included an awareness of DPA performance, the stimulating effect of a daily target goal, and a positive effect on self-efficacy. Motivation dropped when technical problems occurred, which is something that we also encountered in pilot study C3.

### Conclusions

By employing a user-centered design approach, a mobile phone was found to be an adequate and feasible interface for an eHealth intervention because it is non-obtrusive, can measure DPA objectively, and, by using an appropriate app, direct feedback on DPA can be given. Moreover, by combining the app with an appropriate and secured website, monitoring and feedback by an HCP is possible. The mobile phone and app are easy to learn and use by patients with COPD. Battery life lasted a whole day with the final version, and readability and comprehensibility of text and colors were good. The accuracy of DPA measurement was good in the final test. The idea of providing an HCP with DPA data caused no privacy issues in the participants. The final version of the eHealth intervention is presently being tested by our group for efficacy in a RCT in COPD patients.

## Appendix

Multimedia Appendix 1 Results from the consultation rounds. Data from [28,40,41,42].

## Abbreviations

COPD: chronic obstructive pulmonary disease
DPA: daily physical activity
FSU: Florida State University
GOLD: The Global Initiative for Chronic Obstructive Lung Disease
HCP: health care professional
MRC: Medical Research Council
PR: pulmonary rehabilitation
RCT: randomized controlled trial
UMC: University Medical Center
USE: Usefulness, Satisfaction, and Ease of Use questionnaire
