# Supplementary material for: A Mobile Phone App to Stimulate Daily Physical Activity in Patients with Chronic Obstructive Pulmonary Disease: Development, Feasibility, and Pilot Studies
Source: JMIR Mhealth Uhealth. 2016 Jan 26;4(1):e11. doi: 10.2196/mhealth.4741 (PMC4748139; doi:10.2196/mhealth.4741)
Supplement: Multimedia Appendix 1 [file mhealth_v4i1e11_app1.pdf]

	Pilot study	Feedback	Adjustments made
**Use**			
**Mobile phone**			
	C1,3	Wearing the phone all day was not always practical, but because of the benefits it was not seen as problematic.	
	C1,2	Two subjects considered the mobile phone to be quite large and preferred to carry it in their shirt pocket; seven preferred to carry it in their trouser pocket.	Not adjusted because better measurements are achieved by positioning the accelerometer as close to the center of gravity as possible [40,41].
	C1,2	Seven subjects found the phone pouch uncomfortable and did not like its appearance. Putting the mobile phone in the pouch was difficult.	Different phone pouches were acquired.
	C2,3	Use of the touchscreen did not pose any problems in general, although it did for several subjects.	Personal instructions were added to the protocol.
	C1	Battery life was a problem. The mobile phone had to be charged in the afternoon.	Continuous measurement was adjusted to include five minutes of measurement followed by five minutes of no measurement. Data were then multiplied by two to obtain an estimate of total daily steps. This accounted for diminished measurement accuracy but provided day-long battery life, which was deemed necessary for adherence to the intervention.

**Application**			
	C1,2	Learning how to use the app and navigation were found to be quite easy.	An explanatory pop-up screen that appeared when a small question mark icon was touched was added.
	C3	One subject lost interest after one week, but all of the remaining subjects found the app useful and stimulating.	
	C2	The messages helped to maintain interest.	
	C2,3	Reaching their DPA goal was rewarding.	
	C3	Comparing DPA goal progress was fun.	
	C1-3	The reported time points for looking at the app included during a moment of rest, when the phone was being used for other purposes, immediately following an activity and before going to bed.	
	C1-3	Sounds were not desired except for incoming text messages and a reminder to charge the phone.	
	C1-3	Subjects were willing to pay 10-25 euros a month for a subscription to the app that included monitoring from a physiotherapist.	
	C2,3	Reasons to not acquire the app included being confronted with incapacity, that sufficient DPA advice was received, and the need to buy a mobile phone. The main reason for using the app was for health maintenance.	
**Design**			

**Version 1 (Figure 2)**			
	C1	The activity and intensity axes in the dartboard were confusing.	The dartboard was changed to include two bars: steps and intensity.
	C1	The app only provided information on current DPA status, and a cumulative approach would be preferable.	Added
	C1	DPA over time would be a positive addition.	Added
	C1	The wording of the advice was clear.	
**Version 2 (Figure 3)**			
	C2	Subjects wanted to keep the open circles in the filled green circles at all times. It worked like a game and they found it fun.	
	C2	If an emoticon was to be added, they preferred it to be a dog because they had one at home.	The widget was given traffic light colors, and an emoticon with an expression corresponding to current DPA status was added. A dog was the default but other options could be chosen.
	C2	The graph was quite clear; however, the blue line was difficult to see.	The colors yellow and blue were no longer incorporated in the app because elderly users have low perception ability for these colors. [28].
	C2	They suggested the addition of the absolute number of steps taken.	Added
**Version 3 (Figure 4)**			

	C3	Fun and stimulating advice and emoticons.	
	C3	A few mismatches between the advice given and the status of the activity bar, and some pieces of advice were not totally comprehended.	Unclear advice was adjusted, and mismatches were eliminated.
	C3	Readability of the advice could be better.	Contrast was improved, and font size increased.
**Privacy**			
	C1-3	The user should be able to determine who can see their DPA data.	
	C1-3	HCP, family and friends posed no problem.	
	C1-3	With respect to local government and insurance companies, answers varied.	
**Measure-ment of DPA**			
	C1	Unclear what aspects of DPA were being measured.	DPA goals were divided into 5 levels, which were computed based on the results from a previously conducted literature review [42], which incorporated number of steps as well as intensity of DPA.
	C1-3	Subjects regretted that cycling, distance travelled, and intensity of strength training and stair walking were not fully captured by the app.	
	C2	A good way to get acquainted with your activity pattern.	The levels were discarded, and number of steps, minutes and number of steps for an intensive

			minute could be personalized on the website by the HCP.
